# Facilitating physical activity and reducing symptoms in patients with knee osteoarthritis: study protocol of a randomized controlled trial to test a theory-based PrevOP-psychological adherence program (PrevOP-PAP)

**DOI:** 10.1186/s12891-018-2158-8

**Published:** 2018-07-18

**Authors:** Nina Knoll, Diana Hilda Hohl, Susannah Motter, Jan Keller, Daniela Lange, Dieter Felsenberg, Peter Martus, Wolfgang Ertel, Ralf Schwarzer

**Affiliations:** 1 Department of Education and Psychology, Health Psychology Division, Freie Universität Berlin, Habelschwerdter Allee 45, 14195 Berlin, Germany; 2 Centre for Muscle- and Bone Research, Department of Traumatology and Reconstructive Surgery, Charité – Universitätsmedizin Berlin, Hindenburgdamm 30, 12200 Berlin, Germany; 3 Institute for Clinical Epidemiology and Applied Biometry, Universitätsklinikum Tübingen, Silcherstr. 5, 72076 Tübingen, Germany; 4 Department of Traumatology and Reconstructive Surgery, Charité – Universitätsmedizin Berlin, Hindenburgdamm 30, 12200 Berlin, Germany; 5 SWPS University of Social Sciences and Humanities, ul. Ostrowskiego 30b, 53-238 Wrocław, Poland

**Keywords:** Knee osteoarthritis, Physical activity, Osteoarthritis symptoms, Planning, Action control, Social network, Social support, Social control, HAPA, RCT

## Abstract

**Background:**

The present randomized controlled trial, which is crossed with the “PREVenting the impairment of primary Osteoarthritis by high impact long-term Physical exercise regimen” Main Medical Trial (PrevOP-MMT), aims to evaluate a psychological adherence program (PrevOP-PAP), and is designed to support persons with knee osteoarthritis (OAK) in the uptake and maintenance of regular physical activity to reduce OAK symptoms. The PrevOP-PAP is based on the Health Action Process Approach (HAPA), a social-cognitive theory predicting health behavior change in individuals, extended here by social network characteristics and social exchange processes. It is expected that participants with OAK receiving the PrevOP-PAP will maintain higher levels of regular physical activity throughout a 24-month period and consequently report lower levels of OAK symptoms than participants of an active control condition.

**Methods:**

A total of *N* = 240 participants with medically verified moderate OAK will be randomly assigned to an intervention condition (PrevOP-PAP-I; 50%) or an active control condition (PrevOP-PAP-CTRL). The PrevOP-PAP-I includes a motivational intervention, repeated self-regulation interventions, and a network creation intervention delivered over 12 months. Modes of intervention delivery include a paper-pencil motivation leaflet with a quiz, a computer-assisted face-to-face intervention, four computer assisted phone-based interventions, and activity calendars. The PrevOP-PAP-CTRL includes the motivational intervention only. Primary outcome will be OAK symptoms. Secondary outcomes include objectively and subjectively measured physical activity and indicators of quality of life. Other outcomes are HAPA-derived self-regulatory indicators as well as proposed social network and social exchange mechanisms of health behavior change. Assessments take place at baseline, 6 months, 12 months, 18 months, and 24 months following baseline.

**Discussion:**

Based on the extended HAPA, this study seeks to reveal the self-regulatory and social mechanisms of the uptake and maintenance of physical activity and their relation to disease symptoms in persons with OAK. The design and evaluation of this program are intended to become a yardstick for future development and implementation of digitalized psychological adherence programs in this population.

**Trial registration:**

German Clinical Trials Register; also available at http://apps.who.int/trialsearch/; registration number: DRKS00009677; date of registration: 26 January 2016.

## Background

The main objectives for physical activity (PA) prescribed to persons with osteoarthritis of the knee (OAK) are enhancing the mobility and required range of motion of the joint, increasing the force and endurance of the muscles, and reducing pain [1, 2]. Guidelines advise persons with OAK to engage in joint-friendly regular moderate to vigorous PA (aerobic; moderate: at least 150 min per week, or vigorous: at least 75 min per week, or a mixture of both, on at least five days a week, best performed in bouts of at least 10 min) and regular muscle-force strengthening exercises [3]. Such regular PA requires self-regulated action and, therefore, becomes subject to adherence failure [4, 5]. There is no medical solution to this problem, but a psychological adherence program teaching self-regulation strategies to support uptake and maintenance of regular PA may make a substantial contribution [4, 6–8].

**Fig. 1:**
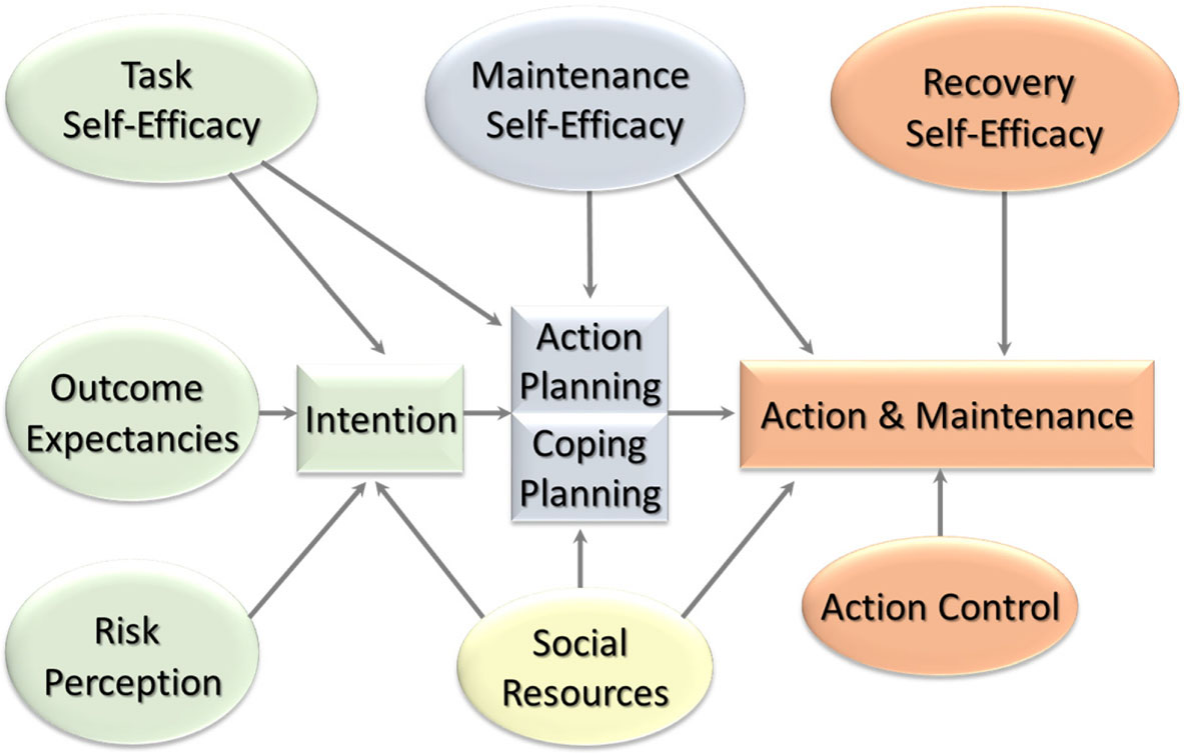
The Health Action Process Approach (HAPA; [16])

Health psychology research has identified various self-regulation strategies to facilitate motivation, to prevent relapse, and to support PA maintenance, based on theory and empirical evidence [9–15]. The present project uses the Health Action Process Approach (HAPA; Fig. 1) as a theoretical backdrop that includes factors predicting intention formation (i.e., motivational stage) and specifies a mediating sequence of factors leading up to the implementation and maintenance of action (volitional stage), in this case, regular PA [16]. The HAPA is a hybrid model of behavior change that is derived from several preceding social cognitive models [17–21]. In the motivational stage, three factors are proposed to strengthen intention formation to change health-relevant behaviors: task self-efficacy or the belief that one is capable of performing the behavior in question, outcome expectancies or a decisional balance of pros and cons associated with behavior change, and risk perception regarding health consequences if behavior is not changed. Several other self-regulatory factors are then proposed to mediate between intention and behavior in a volitional stage, including planning, maintenance self-efficacy, recovery self-efficacy, and action control [16]. Planning contains strategies that help the individual to implement behavior by a-priori linking situational cues with the intended behavior change (action planning) and strategies that additionally facilitate risk management by anticipating barriers and linking them with planned behavioral alternatives (coping planning). Maintenance self-efficacy and recovery self-efficacy refer to the subjective belief that one is capable to maintain behavior change for an extended amount of time or resume it once a lapse has occurred, respectively. Action control is composed of three subfacets including awareness of standards or intentions, self-monitoring of the actual behavioral response, and regulatory efforts should the current behavior diverge from what was intended [16, 22]. Additional resources of behavior change, such as use of social exchange strategies (e.g., social support, see below) are also suggested to impact intention formation and behavior (Fig. 1).

Respective theory-based intervention strategies to facilitate adoption and maintenance of PA include intention formation by goal setting, inducing positive outcome expectancies, and task self-efficacy as well as volitional strategies that guide participants in their formulation of action plans, coping plans, and practice of continuous action control [10, 14, 23]. Evidence from HAPA intervention studies yields results for populations with various chronic illnesses and disabilities [14]. Especially planning interventions and action control programs proved to be successful. Evidence has emerged in the context of orthopedic and cardiac rehabilitation [10, 22, 24]. Randomized controlled trials have documented the evidence in favor of planning interventions [25, 26]. When addressing action planning and coping planning separately in interventions, different effects were found: Persons in cardiac rehabilitation became more active when both kinds of planning were addressed in the intervention, as opposed to a mere action planning intervention [27]. In orthopedic interventions, coping planning seemed to be of particularly high importance for long-term maintenance [10]. Self-monitoring as part of action control is an essential behavior change technique that can be applied to various health behaviors. When people keep records of their behaviors in form of a diary, checkmarks in their calendar, or by using electronic messaging, they become aware of gains and deficits in the implementation of behavior change which allows them to take further regulatory action [22, 23]. In addition to intensive action control regimens, a specific strategy to prevent relapse into sedentary behavior includes booster sessions [10, 24]. Follow-up intervention boosters refer to brief contacts beyond the main part of the intervention to reinforce previous intervention content [10]. Adding booster sessions to an initial face-to-face intervention and thus supporting the maintenance of self-regulation strategies can be one way to achieve behavior maintenance. In the clinical context, telephone-delivered intervention boosters have been successfully implemented to promote exercise maintenance [10, 24].

As persons’ close social network members (e.g., spouses, family, sports companions) often try to and succeed in co-regulating an actor’s health behavior, an extension of core predictors of individual health behavior change to include network formation and emergent social exchange strategies with important others is called for [28–30]. Recent findings suggest that by including another person into the behavior change process, e.g. by dyadically planning an individual actor’s health behavior change together with a planning partner, a number of potentially beneficial processes are triggered [12, 31, 32]. These include enhanced self-regulatory action control by the actor, but also a number of social exchange processes initiated by the planning partner that may help the actor maintain behavior change, including social support and social control [31, 32]. Social support refers to assistance from others that may help a recipient achieve specific behavioral goals [33, 34]. Social control, on the other hand, refers to strategies that aim at influencing or regulating another person’s behavior, such as direct persuasion, pressure, nagging or positive reinforcement of desired behavior [35]. Whereas social control may be a double-edged sword with the capacity of supporting, but also compromising the behavior change process [36, 37], it is likely to be triggered in interventions addressing network formation and therefore needs to be accounted for. In accord with Bodenmann’s cascade model [38], social exchange strategies may be especially effective in the long run, when initial self-regulation starts to wear out [39]. Moreover, social exchange strategies were shown to be prospectively associated with several self-regulatory processes reviewed above and may thus serve as a real-life booster of them [30, 36].

Although a vast amount of research on physical activity in persons with OAK has already been performed, most of it is not theory guided [1, 2, 40], and therefore, innovative theory-based programs for exercise maintenance are needed. In particular, the roles of action control, network formation, and social exchange strategies that support and maintain self-regulatory processes over a longer period of time need further investigation. Therefore, the present research design includes multiple treatment occasions and measurement points over an extended period of two years.

### Aims and primary research question

The present randomized controlled trial (RCT) is crossed with the “PREVenting the impairment of primary Osteoarthritis by high impact long-term Physical exercise regimen (PrevOP)”-Main Medical Trial (henceforth referred to as PrevOP-MMT; see Armbrecht et al. Preventing the impairment of primary osteoarthritis by high impact long-term physical exercise regimen (PrevOP): Study protocol of a randomized controlled trial. Manuscript in preparation.). Using the HAPA [16] as a theoretical backdrop and extending it with network characteristics and emergent social exchange processes that are proposed to facilitate behavior change, this project develops and evaluates a theory-based PrevOP-Psychological Adherence Program (henceforth referred to as PrevOP-PAP). The PrevOP-PAP is designed to strengthen long-term self-regulation in the adoption and maintenance of PA by using regular boosters of main intervention contents and by facilitating social network creation that is proposed to result in the additional social exchange processes supporting the maintenance of regular PA. The initiation and maintenance of regular PA is in turn proposed to be a behavioral predictor of reduced OAK symptoms (as assessed by the Western Ontario and Mc Master Universities Osteoarthritis Index [WOMAC]). Because the PrevOP-PAP intervention addresses predominantly volitional factors, the efficacy of the intervention condition will be compared to an active control condition; both conditions involve a brief motivational treatment [16].

#### Primary research question

Compared to an active control condition, can a theory-based psychological adherence program designed to increase self-regulation and network formation facilitate the uptake and maintenance of regular PA (key secondary outcome) and contribute to a reduction in symptom severity (primary outcome) in persons with moderate OAK?

## Methods

### Design

**Fig. 2:**
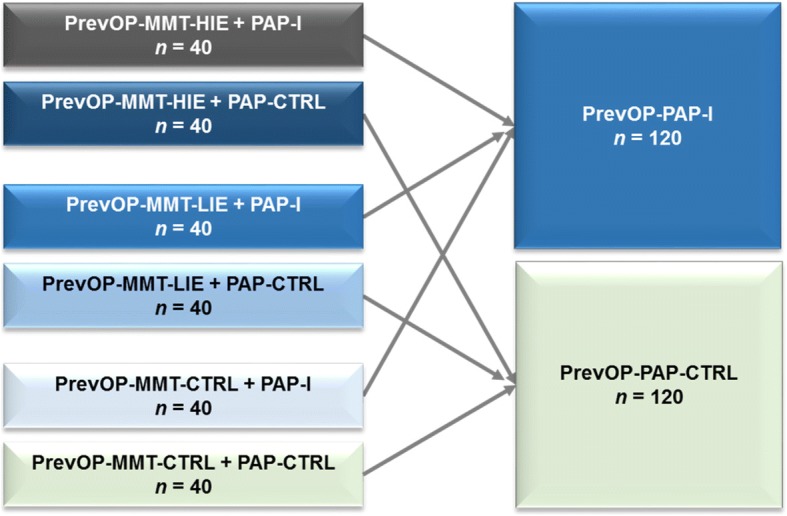
Conditions of the PrevOP-Main Medical Trial (PrevOP-MMT;) nested in the PrevOP-Psychological Adherence Program (PrevOP-PAP) conditions (PrevOP-PAP-I and PrevOP-PAP-CTRL)

The PrevOP-PAP trial shares participants, inclusion-, and exclusion criteria with the PrevOP-MMT (crossed design). In the PrevOP-MMT, participants with OAK (*N* = 240), a randomized 33% of whom receiving a high impact long-term physical exercise regimen (PrevOP-MMT-HIE) with resistive vibration exercise, 33% receiving a low-impact long-term exercise regimen (PrevOP-MMT-LIE) and 33% receiving no structured or monitored exercise regimen (PrevOP-MMT-CTRL;), are again randomly subdivided into 50% who undergo the PrevOP-PAP intervention (PrevOP-PAP-I, *n* = 120) and another 50% who serve as active controls (PrevOP-PAP-CTRL, *n* = 120). Forty participants are thus randomly assigned to each of 6 (3 PrevOP-MMT * 2 PrevOP-PAP conditions) study conditions and then collapsed into either the PrevOP-PAP-I (*n* = 120) or the PrevOP-PAP-CTRL (*n* = 120) (see Fig. 2).

**Fig. 3:**
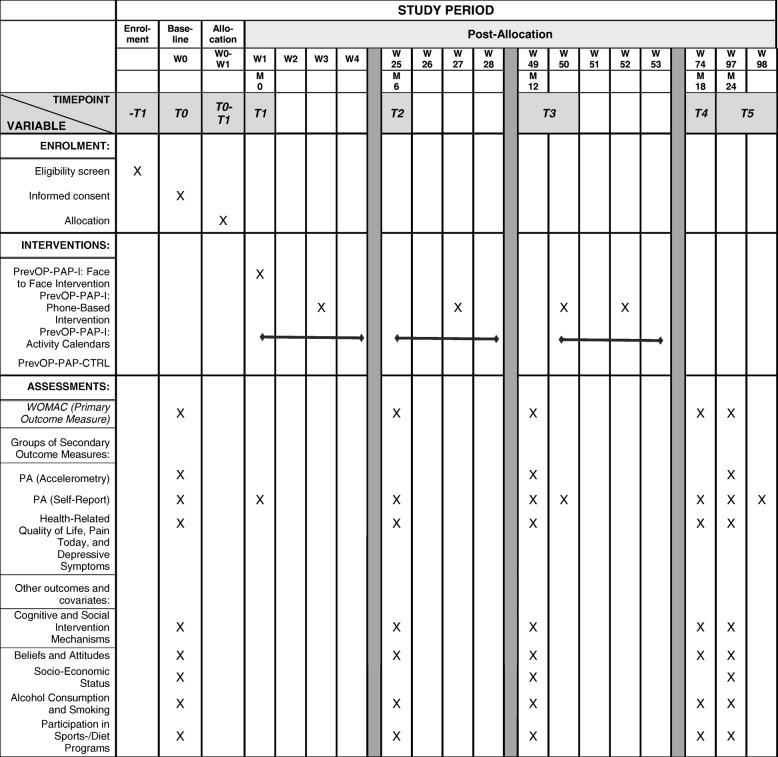
SPIRIT chart of the PrevOP-PAP trial. W: Week. M: Month. T: Time. PrevOP-PAP-I: PrevOP Psychological Adherence Program Intervention condition. PrevOP-PAP-CTRL: Psychological Adherence Program ConTRoL condition

Data are assessed at baseline (T0), 6 months (T2), 12 months (T3), 18 months (T4), and 24 months (T5) via self-report measures and three one-week assessment periods (T0, T3, T5) when participants wear accelerometers for one-week periods each. Within the PrevOP-PAP-I, the main computer-assisted face-to-face intervention takes place at T1 (one week following T0), additional action control and booster intervention phases take place between week 1 and week 4, between week 25 and week 28, and between week 50 and week 53 following baseline (T0), using computer-assisted phone-based interventions and paper-pencil activity calendars (i.e., action control intervention, see below). Initial self-report assessments and the computer-assisted face-to-face intervention are done at the study center at Charité – Universitätsmedizin Berlin. Accelerometers are worn for one-week periods in daily life during wake time. For computer-assisted phone-based interventions, participants are called at home. Activity calendars are completed daily at participants’ homes for three periods of four weeks each (see Fig. 3). Participants receive a lump-sum travel cost reimbursement of EUR 5 per study center visit (assessment or intervention sessions). The ethics committee of the Charité – Universitätsmedizin Berlin approved this study (EA4/027/15).

### Sample and recruitment

A total of 240 persons with OAK aged between 40 and 80 years will be recruited for participation in all parts of the study (i.e., PrevOP-MMT and PrevOP-PAP) by the PrevOP-MMT study staff. Reactive recruitment strategies will include flyers, social media, press-releases, and interviews asking those interested to contact the study center. Proactive recruitment strategies will include mailings in stratified age and gender groups as determined by local registration offices and recruitment of patients from the project-associated ambulatory clinic. Inclusion and exclusion criteria are relevant mostly for the PrevOP-MMT. They are as follows:

#### Inclusion criteria

Ambulatory men and women aged between 40 and 80 years with OAK grades 2 and 3 following the Kellgren and Lawrence classification [41], according to American College of Rheumatology criteria [42] with pain on at least half of the days of the previous month (intensity > 40 mm on a 100 mm visual analogue scale). Radiographical signs will include Kellgren and Lawrence grade 2 (i.e., definite osteophytes and possible narrowing of joint space) or grade 3 (i.e., moderate multiple osteophytes, definite narrowing of joint space and some sclerosis, and possible deformity of bone ends), and joint space width of 2.5 to 5 mm with predominant knee osteoarthritis of the medial tibiofemoral compartment, written informed consent.

#### Exclusion criteria

Knee total endoprothesis at any time, hip total endoprothesis during the last 12 months, participation in other clinical trials, cognitive impairment, insufficient comprehension of the German language. During the past 5 years: cancer, angina pectoris, coronary intervention, thromboembolic events (acute deep venous thrombosis, pulmonary embolism, stroke, myocardial infarction, arrhythmia absoluta, uncontrolled hypertension, retinal artery occlusion, vena centralise retinae occlusion). Paraplegia, hemiplegia, severe rheumatoid arthritis, severe polyneuropathy, severe vertebral disc prolapse, severe spinal canal stenosis, dementia, acute tendinitis (legs only), current fractures (pelvic and legs), cholecystolithiasis, kidney stones, urethral stones, hernia abdominalis and/or inguinalis, acute migraine, current wounds (pelvis and legs), epilepsy, pregnancy. Recent intra-articular injection (notably glucocorticoids < 3 months previously or hyaluronic acid < 6 months previously), clinical deformities, and previous treatments acting on cartilage, oral glucosamine ≥1500 mg/day and chondroitin sulphate < 3 months previously).

Inclusion and exclusion criteria are checked by the PrevOP-MMT’s medical personnel during a first screening telephone interview and a subsequent appointment for extensive medical screening assessments at the study center. Informed consent and inclusion into the study takes place at the beginning of baseline assessments (T0) and is done by the PrevOP-MMT’s medical personnel, when participants receive extensive information on the study procedure (PrevOP-MMT and PrevOP-PAP parts; oral and written) including randomization procedures, provide written informed consent, and then begin their first period of assessments.

### Power analysis

For the PrevOP-MMT a sample size of *N* = 240 has been determined. For the PrevOP-PAP, choosing an alpha level of .05 and a stability factor of .68 of the WOMAC (www.rheumatology.org), a minimum sample size of *n* = 122 is required which includes 2 groups at 5 points in time up to the primary endpoint (baseline to 24 months) to detect a small effect (*f* = 0.1) of a within by between subjects factors interaction with a power of .95. With an expected drop-out rate of 20%, the required sample size increases to *n* = 153. This required sample size for the PrevOP-PAP trial is thus in accord with the PrevOP-MMT-required inclusion of *N* = 240 participants.

### Randomization

Randomization will take place on an individual level. Before providing informed consent, participants are fully informed about the trial’s randomization procedures. Following inclusion, i.e., between T0 and T1, participants will be randomized to one of 6 study arms (3 PrevOP-MMT * 2 PrevOP-PAP conditions, see Fig. 2), using computer-generated random numbers, stratified by gender. Randomization procedures take place outside the study center at the Institute for Clinical Epidemiology and Applied Biometry at Tübingen University Medical Center. PrevOP-PAP study staff are informed about participant allocation to intervention arms via fax by the end of the baseline phase.

### Masking

Regarding study staff conducting the PrevOP-PAP intervention sessions, allocation of participants to intervention or control conditions cannot be masked. Study staff know participants’ allocation at the beginning of the face-to-face computer-assisted intervention session (T1). Moreover, allocation to study condition cannot be masked for participants, as upon inclusion, they are provided a-priori information on the existence of different PrevOP-PAP conditions and randomized allocation to one of them. Analyses of data will be conducted by PrevOP-PAP study staff and will not be masked.

### Brief motivation treatment for all participants

A brief motivational intervention will be delivered to all participants via printed material containing an information leaflet that participants will be asked to read and a brief quiz to test recollection of the information conveyed via the leaflet.

The motivation treatment leaflet contains information on increasing PA for persons with OAK, introduces different intensities of PA and PA guidelines for persons with OAK, and addresses outcome expectancies, risk perceptions, self-efficacy beliefs, and role models related to PA [3, 17, 43]. Below, associated behavior change techniques (BCTs; [13]) are provided in brackets, following the description of text provided in the leaflet. The leaflet is printed in color on a single Din-A 4 page (front and back) that is folded twice and thus includes 6 individual pages. Throughout the leaflet, text passages are interspersed with photos suggesting success in PA, depicting sports utensils, framed summary messages, and illustrations meant to underscore the topic of the respective section. The title page of the folded leaflet informs about the project title and depicts the study logo, the logos of the collaborating institutions, as well as a photograph of a walker’s walking calves and sneaker-clad feet.

The second page starts with a brief basic description of the etiology, risk factors, symptoms, and functional limitations associated with OAK [44] and closes with a statement that evidence indicates that regular joint-friendly activity can help to slow the progression of OAK [3, 44, 45]. This section is interspersed with brief framed calls for PA. On the third page, activity guidelines for persons with OAK are stated, activities of different intensities and types (moderate and vigorous, as well as muscle-force strengthening exercises) are explained and examples of joint-friendly moderate activities and muscle-force strengthening exercises are given (BCTs 1.1, 4.1, 9.1; [3, 13]).

The fourth and fifth pages introduce generic and OAK-specific benefits of daily PA, followed by a list of risks of insufficient activity, and often anticipated negative outcomes of PA. Following this, a brief introduction of the role of negative and positive outcome expectancies for motivation is presented (BCTs 5.1, 5.6; [13]). The final page addresses the participants’ social environment. Participants are asked to reflect what their social network would think of them becoming more active (BCT 3.1; 5.3; [13]) and think about PA role models in their network. This then leads to a brief section on the role of self-efficacy for motivation, asking participants to use self-instruction and exploit former mastery experiences for an increase in PA-specific self-efficacy (BCTs 10.4, 15.3, 15.4, 16.3; [13]).

The *quiz* is delivered in form of a cross-word puzzle. Participants are asked to complete 8 (partially solved) fill-in-the-blanks statements on subject matters introduced by the motivation leaflet and then transfer the filled-in terms to a cross-word puzzle with a pen. The backside of the quiz sheet features the correctly completed cross-word puzzle.

### PrevOP-PAP intervention and -control conditions

The PrevOP-PAP is designed to improve motivation to be physically active and enhance exercise adherence by conveying volitional self-regulation strategies to improve maintenance and prevent relapse to sedentary behavior in persons with OAK.

#### PrevOP-PAP-control condition (PrevOP-PAP-CTRL)

Participants within the PAP-CTRL (*n* = 120) will receive the motivation treatment at T1, but no further psychological interventions. Instead, like the individuals from the PrevOP-PAP-I condition, PAP-CTRL participants receive multiple assessments to monitor changes in primary and secondary outcomes.

#### PrevOP-PAP intervention condition (PrevOP-PAP-I)

Participants (*n* = 120) within the PrevOP-PAP-I will receive a comprehensive intervention program during the first year of study participation (Fig. 3). Motivational interviewing and self-regulation education will take place, which are boostered at regular intervals. The main intervention delivery modes addressing PrevOP-PAP-I components will be computer-assisted face-to-face and phone-based interventions, conducted by trained study staff and paper-pencil materials (PA calendars). The PrevOP-PAP-I is compatible with all PrevOP-MMT conditions in that participants can use self-regulation strategies to maintain all specific PrevOP-MMT-instructed exercise forms (PrevOP-MMT-HIE; PrevOP-MMT-LIE) or self-selected PA (PrevOP-MMT-CTRL). Thus, PrevOP-PAP-I strategies will be parallel across all PrevOP-MMT-conditions.

##### PrevOP-PAP-I computer-assisted face-to-face intervention (week 1 following T0-assessment period; duration about one hour)

PrevOP-PAP-I participants are scheduled to return to the study center at Charité – Universitätsmedizin Berlin following their one-week T0 period. Participants are led to a separate room, take a seat next to the trained study staff and both are guided through the intervention by a computer program seen on the screen of a laptop placed before them. In addition to introductory and feedback sections, the session is divided into four sections focussing on: outcome expectancies, mastery/self-efficacy, goal setting, and planning. Behavior change techniques (BCTs; [13]) used in this part of the intervention are provided in brackets following a brief description of intervention sections described below. The program is presented in a web browser with fill-in options on several consecutive pages. Intervention content-fields are enriched by photos of physically active adults (mid-age to old age), sports utensils, framed summary messages, and depictions meant to illustrate the topic of the respective intervention section.

#### Introduction

The session starts with a reiteration of the project goals (i.e., prevention of OAK progression by means of PA) and the goals of the present intervention session (i.e., develop strategies to implement intentions and overcome barriers). Participants are reminded that PA helps with OAK symptoms, but that regular PA may not be an easy task which is why self-regulation strategies are practiced in this intervention session (BCT 4.1; [13]).

#### Outcome expectancies

Participants calculate their own pros-cons difference of outcomes of regular PA by indicating their agreement with 5 positive (less joint stiffness, good for overall health, enhanced quality of life, stabilize/reduce weight, social contacts) and 5 negative (worsening of symptoms, pain during specific activities, too exhausting, time-consuming, fear of humiliation) outcome expectancy statements on 6-point Likert scales (not at all true – completely true) presented on the computer screen. A score is provided for expected pros and one for expected cons and computed into a benefit expectation difference displayed on the screen. In case of con-scores outweighing pro-scores, participants’ concerns are reviewed and participants are asked to identify activities producing less cons (BCT 9.2; [13]).

#### Mastery/self-efficacy

Working on a PA biography, participants are instructed to recollect types of PA and positive experiences with them throughout their life-span (i.e., childhood/adolescence, early adulthood, middle age; BCT 2.3, 13.1, 13.5; [13]). Activities and experiences are typed into the program by study staff. At the end of this section, an overview of identified PA that were a source of positive experience is provided.

#### Goal setting

This section opens with a reiteration of OAK-specific PA guidelines with explanations for activity types and intensities, and joint-friendly activity examples [3], followed by brief depictions of testimonials by a 61-year old man and a 68-year old woman describing pursuits of their PA goals. Then participants are asked to set and record up to five PA goals (i.e., type of activity, duration) for themselves, they may choose activities they are already performing or set goals for new activities (BCT 1.1; [13]).

#### Planning

Identified PA goals are fed back into the planning section of the intervention. Participants are asked to generate specific action plans for the implementation of their set PA goals. Plans are phrased in an “If/When…, then…” format. Participants are instructed to provide a specific cue situation in the “if/when”-part and describe the planned activity in the “then-”part. An example is provided (“When I come home from work on Wednesdays, then I go aquajogging for 60 minutes”). Then participants are asked to indicate on a 6-point response scale (not at all true to completely true) their agreement with the following statement (plan execution self-efficacy; [46]): “I am sure that I can act as planned in this situation”. Plans are recorded in the computer program. Next, participants are asked to name a start date for the implementation of their plans and copy their plans, as displayed to them on a summary list, into one of the provided activity calendars (see below). Following this, participants are fed back their action plans one-by-one and asked to generate one coping plan for each action plan by (1) thinking about barriers that may keep them from adhering to their action plan and (2) thinking of strategies to manage these barriers (e.g., performing the planned activity at a different time or place and/or performing an alternative activity). Coping plans are also formed in an “If…, then...” format (provided example: “If a spontaneous visit with a friend comes up, then I drive to the friend’s house by bike.”) and all information is recorded. A final summary sheet of all action plans and coping plans is shown (BCT 1.4; [13]).

#### Feedback

A feedback section then asks participants to rate the quality of the session on a 6-point German school-grade scale (very good – good – satisfactory – sufficient – unsatisfactory – insufficient) and provide written additional feedback if they wish.

At the end of the session, participants are asked to regularly complete their activity calendars (see below), an appointment for the following booster session via computer-assisted phone-based intervention is scheduled, participants are thanked and dismissed.

##### PrevOP-PAP-I computer-assisted phone-based interventions (weeks 3, 27, 50, and 52 following T0)

Throughout the following 12 months (Fig. 3), four computer-assisted phone-based interventions are scheduled as booster sessions of the main face-to-face intervention session (T1) described above. Participants are called by study staff at pre-scheduled appointments and are guided through the booster interventions over the phone. Study staff follow a computer-based structured intervention and record participants’ responses in a computer program displayed in a browser. Earlier responses from participants’ face-to-face computer-assisted intervention or earlier computer-assisted phone-based interventions are fed back into the new session. All four phone-based interventions are nearly identical (except for varying time frames since the last intervention session and addenda in phone-based interventions 3 and 4) and designed to boost participants’ planning, self-efficacy, and action control regarding their regular PA. The phone-based interventions 3 and 4 have an additional component of network creation, i.e., during phone-based intervention 3, participants are prompted to find a sports companion to be active with them on a regular basis and during phone-based intervention 4, they are asked to include the sports companion into their action plans. Behavior change techniques (BCTs; [13]) used in phone-based interventions are provided in brackets following a brief description of intervention sections described below.

#### Introduction

Following initial greetings, a brief conversation on how the participant fared since the last contact, and a brief introduction into the purpose and structure of the phone-based intervention session are given. Participants are then asked to fetch activity calendars that they completed during the past two weeks (phone-based interventions 1, 2, and 4) as well as two new activity calendars that have been provided before (see below) as support material for the session. Participants are then generally asked how they fared with their most-recently completed activity calendars and implemented PA goals during the past two weeks (phone-based interventions 1, 2, and 4).

#### Goal/plan review

Following this general assessment of the PA goals for the past two weeks, each goal set during the past intervention session is reviewed. Participants are read their past PA goal and their matching action plan and asked how successful they have been in implementing this plan (in %). Patients are instructed to refer to their past two-week activity calendars to produce an estimated percentage of their goal attainment. They are then asked to recollect positive experiences they have had when implementing their action plan. Then participants are asked whether they want to continue with this particular PA goal and/or action plan for the next weeks or whether they want to change it/them. If participants want to change their PA goal, they are asked which other activity they would like to carry out. They are then asked to form a new action plan, following an “If/When.., then…” structure. Then participants are asked to indicate on a 6-point response scale (not at all true to very true; read to them) their agreement with the following statement (plan execution self-efficacy; [46]): “I am sure that I can act as planned in this situation”. Next, participants are instructed to form a new coping plan for the changed activity goal and/or action plan. Again, coping plans are to be framed in an “If…, then…” structure. Changed activity goals, action plans, plan self-efficacy scores, and coping plans are recorded by study staff. If participants do not want to change activities and/or plans, their past entries are kept in the system, but new plan self-efficacy items are completed. At the end of the review of former goals and plans, participants are asked if they want to add new PA goals to their list (up to a maximum of 5 PA goals all in all), in which case the new PA goal, action plan, plan self-efficacy, and coping plan-procedure is then repeated for as many PA goals as are newly set. Participants are then slowly read each action plan (kept, altered, and new ones) and asked to fill them into their new activity calendars. A list of all action and coping plans generated during this session is printed and sent to participants via mail. Participants are then reminded to keep completing their new activity calendars during the next two weeks (BCTs 1.1, 1.3, 1.5, 1.7; [13]).

#### Phone-based intervention 3 addendum -- network creation

At the beginning of this section which follows the above-described goal/plan review section, participants are asked if they have ever engaged in physical activity together with another person. If participants answer in the affirmative, they are instructed to recollect positive experiences with joint physical activity in the past and are asked whether they could imagine to be physically active with another person in the future as well. If participants affirm, they are asked to indicate the initials and the nature of the relation to this person (e.g., partner, child, friend, neighbour). If participants negate to have ever engaged in physical activity together with another person in the past, they are asked if they can imagine to be physically active with another person in the future. If participants affirm, they are again asked to indicate the initials and the kind of relation they share with this specific person and are instructed to contact this person and ask them if they wanted to be their sports companion before phone-based intervention 4. If participants negate, this issue is not further explored (BCTs 3.2; [13]).

#### Phone-based intervention 4 addendum -- network creation review

Following the introduction section and before the goal/plan review section, participants who have indicated during the phone-based intervention 3 that they wanted to engage in physical activity with another person are reminded of this and asked if this is still the case and whether it is still the same person. If participants do not want to be active with someone, the same goal/plan review section as described above continues. If participants respond in the affirmative or in case participants now name a new person as a potential sports companion, then this issue is taken up in all following goal/plan reviews. During each goal/plan review, participants are asked to indicate if they want to include their sports companion in this action plan and indicate the sports companion’s initials and in which type of relation they are. Participants are then asked to include in the “then...”-parts of their “If/When…, then...” action plans the persons with whom they want to be active. All information is recorded by study staff (BCTs 1.4, 3.2; [13]).

#### Feedback

A feedback section then again asks participants to rate the quality of the session on a 6-point German school-grade scale (very good – good--satisfactory – sufficient—unsatisfactory – insufficient) and provide additional oral feedback if they wish. Feedback scores and points are recorded by study staff.

At the end of each phone-based intervention-session that lasts between 20 and 60 min, participants are asked if they have any questions, reminded of the next study appointment, reminded to use the learned self-regulatory strategies in their daily lives, and thanked.

##### Activity calendars (weeks 1 to 4, weeks 25 to 28, weeks 50 to 53 following T0)

Participants are provided with printed sets of activity calendars to be taken home and completed daily at the end of the day throughout weeks 1 to 4 (following their face-to-face intervention and phone-based intervention 1), weeks 25 to 28 (surrounding phone-based intervention 2), and weeks 50 to 53 (following phone-based intervention 3 and interspersed with phone-based intervention 4) following T0 (Fig. 3). Activity calendars provide written instructions on the top and are depicted in form of tables with columns for 7 days. Columns and column headers are blank for participants to fill in the date and days of the week in column headers and their created action plans for each day during the face-to-face or phone-based interventions. Filled-in plans can be abbreviated, but shall minimally contain the situational cues to trigger action, what participants are planning to do, and for how long (e.g., Tuesday-column: “after breakfast, go swimming, 25 minutes”). Next to each day column there is a narrower column where participants are instructed to put a checkmark next to their action plan if they have implemented their plan on a given day. At the bottom of the calendar section there is an additional field where participants can note additional PA bouts pursued during a given day. All calendars and their instructions are identical except for the last two ones to be completed following phone-based intervention 4 (from week 52 following T0). Here, participants are additionally instructed to write down with whom they plan to pursue the planned activity (e.g., after lunch, ride my bike at a good pace, 40 min, with my husband). Calendars are meant to trigger participants’ action control and support their development of self-efficacy by daily review of their mastery experiences. Following the completion of four calendars in a row, participants are asked to either hand them to or mail them back to study personnel, participant codes are noted on the top of each calendar sheet (BCT 2.3, 2.4, 2.7; [13]).

### Benefits and harms

Participants in the PrevOP-PAP-I may benefit from the use of self-regulatory strategies taught in the intervention not only in terms of the uptake and maintenance of regular PA, but also in terms of other volitional activities of daily living (e.g., eating, chores, social activities). Transfer of the use of these strategies is encouraged in the interaction with study staff during intervention sessions. If participants manage to maintain regular PA, they may experience a decrease in OAK symptom severity as well. The PrevOP-PAP-I is not expected to be harmful for participants which is why a data monitoring and safety board or stopping rules are not needed for the PrevOP-PAP trial. Due to data-protection principles, outcomes of the psychological screening instrument Center for Epidemiologic Studies Depression Scale are not individually monitored (see below; note that this version of the scale does not contain an item on suicidal ideation) [47]. Instead, all participants are routinely given information about emergency services they can turn to should they experience psychological problems.

### Data collection and entry

Data are collected over a 2-year period (i.e., participants remain in the study for 2 years) at 6 measurement points (or periods) in time (T0 to T5) by trained study personnel (see Fig. 3). Inter-measurement intervals range from daily to 6 months, depending on the assessment in question. Data are assessed with self-report questionnaires and objective measures (e.g., accelerometers, body weight). Participants schedule assessment sessions at the study center at Charité – Universitätsmedizin Berlin. During assessment sessions, they are handed a questionnaire booklet, asked to complete it during the session and instructed to turn to study staff with questions (T0, T2, T3, T4, T5; T1 is the main intervention session with additional assessments). At T0, T3, and T5 participants undergo further medical examinations and are handed and instructed to wear accelerometers for eight days. At the end of T0, T3, and T5 periods, i.e., following each one-week accelerometer assessment, participants are additionally asked to complete a brief self-report measure on their daily PA during the past week. Completed study materials and accelerometers are either directly returned to study personnel or mailed to the health psychology lab at Freie Universität Berlin. Data entry will be conducted by trained PrevOP-PAP study personnel. Quality management of data entry will include guidance by a data entry manual, regular plausibility checks, and double entry of portions of the assessed data.

#### Primary outcome: OAK symptoms

PrevOP-PAP’s primary outcome are self-reported OAK symptoms assessed with the Western Ontario and Mc Master Universities Osteoarthritis Index (WOMAC; [48]), in its version for OAK. The WOMAC contains 24 items on symptoms of OAK including stiffness, pain, and functional limitations. Participants are asked to respond to items on 11-point numeric rating response scales anchored at “no pain” (0; stiffness or limitation, respectively) and “extreme pain” (10; stiffness or limitation, respectively). The WOMAC has been reported to be reliable and valid [48].

#### Secondary outcomes

##### Physical activity (PA)

Daily PA (in minutes) is measured with tri-axial accelerometer devices (ActiGraph, GT3X) providing information on activity counts. During each assessment period (see Fig. 3), participants are instructed to wear devices strapped around their hips for 8 days, during their waking hours. As devices do not have screens, participants are unable to monitor their PA data on their own. Following assessment periods, data are downloaded from the devices by trained study staff. Using a software program, activity data are then grouped into minutes of PA of different intensities; i.e., light activity, moderate to vigorous physical activity (MVPA), and sedentary behaviors. Data are to be used if participants have worn accelerometers on at least 4 days for at least 10 h a day. In addition to objective assessments of PA, also repeated self-report assessments are conducted using the German version of the Office in Motion Questionnaire ([49]; see Fig. 3) which is complemented by household and work-domain activities as assessed with its predecessor measure (Physical Activity Questionnaire; [50]). The Office in Motion Questionnaire assesses the duration of daily PA of different intensities in the domains of transportation (at work/outside of work), household, work, leisure time, and sports activities during the past week. A sum score of minutes spent in activities of different intensities will be generated. Authors of the instruments reported satisfactory reliability, validity, and change sensitivity for the measures [49, 50].

##### Health-related quality of life and depressive symptoms

Generic health-related quality of life is assessed with a 12-item measure [51]. Additionally, a 1-item visual analogue scale asks participants to rate their pain on the present day [52]. Depressive symptoms are assessed with the 20-item German version of the Center for Epidemiologic Studies Depression Scale [47].

#### Other outcomes and covariates

##### Cognitive and social intervention mechanisms

Most components of intervention mechanisms are derived from the HAPA and serve as further outcomes. Scales are PA-specific and were adapted from Sniehotta et al. (2006) [27], where they showed satisfactory internal consistencies and validity indicators. Unless otherwise noted, response options range from 1 = “does not apply at all”/“very unlikely” to 6 = “applies exactly”/“highly likely”. Motivational intervention mediators include: Risk perceptions (3 items), task self-efficacy (4 items), outcome expectancies (6 items positive, 4 items negative), and intention to be physically active (4 items). Volitional intervention mediators include: Action planning (4 items), coping planning (5 items), maintenance self-efficacy (3 items), recovery self-efficacy (3 items), and action control (6 items).

A network-creation index was modelled after the HAPA’s stage algorithm [27]. Participants are asked to indicate the response that fits them best: (1) I have not yet thought about exercising with someone else (2) I have thought about exercising with someone else, (3) I have thought about exercising with someone else and am looking into this soon, (4) I have already looked for sports companions, but so far have not found one. (5) Right now, I am exercising with someone. (6) Right now, I am not exercising with anyone any longer. If participants are active with other persons, they are asked to indicate their relations with them and identify them by their initials. Additionally, collaborative action planning (3 items) and collaborative coping planning (3 items) with potential sports companions are assessed. Items are modelled after those of individual planning, only they refer to the target person and the sports companion.

Additional social exchange strategies with important or close others, that may or may not be identical with the sports companion are also assessed. Before participants respond to social exchange strategy items, they are asked to indicate the relationship to and initials of the important or close person they refer to (“significant other”). Social exchange strategy items were adapted from Burkert et al. (2011) and Knoll et al. (2017) [31, 32], where they showed satisfactory psychometric properties. All assessed information refers to the identified significant other as a source or provider of social exchange and is specific to the domain of PA: Dyadic action planning (4 items), dyadic coping planning (5 items), mobilized social support (4 items), received social support (6 items), and received social control (6 items). Response options range from 1 = “does not apply at all”/“very unlikely” to 6 = “applies exactly”/“highly likely”.

##### Beliefs and attitudes

A number of self-regulatory beliefs are also assessed. Sources of self-efficacy are assessed with a scale by Warner et al. (2014) [53]. This scale assesses mastery experiences (2 items), vicarious experiences (2 items), symbolic experiences (4 items), and physiological states (4 items) [17]. Additionally, a 3-item measure of attitudes towards exercising with others was developed. Exercise autonomy (4 items), competence (4 items) and relatedness (4 items) is assessed with a measure by Vlachopoulos and Michailidou (2006) [54]. Beliefs about willpower are assessed with a 12-item measure by Job, Walton, Bernecker, and Dweck (2013) [55]. Again, response options range between 1 = “does not apply at all” to 6 = “applies exactly”.

##### Additional information and socio-economic variables

Additional information about participation in other PA programs or sports clubs (3 items), dieting (1 item; “Are you currently on a diet to lose weight?”) and/or participation in commercial diet programs (3 items) is assessed by asking patients whether this is the case (yes/no), which program they attend, and for how long they have been attending (years and months). Alcohol consumption is assessed by asking participants to estimate the number of standard servings of alcohol consumed per week. Smoking is assessed by four items adapted from Ochsner et al. (2015) [56]. Height and weight are assessed objectively as part of the medical examination in the PrevOP-MMT. Additionally, the following socio-economic variables are assessed: Sex, age, marital status, number of children, living arrangements (alone vs. with others), school education, professional training, employment status, monthly net income, nationality, and native language.

### Statistical analyses

#### Randomization check, manipulation check, drop-out analyses, and intention to treat

A randomization check will address equal distributions of all baseline measures of all outcomes and covariates across conditions using multivariate analyses of variance and chi-square tests for nominal and ordinal-scale baseline data. Manipulation checks of intervention strategies will test differential intra-individual increases of cognitive and social variables targeted with the different intervention modules. For this, 2-level mixed models with TIME as a within-subjects factor and condition (PrevOP-PAP-I; PrevOP-PAP-CTRL) as the between-subjects factor as well as their interaction will be fit.

Analyses will be carried out in an intention-to-treat manner. Depending on the analytical approach to the main hypotheses, multiple imputation or full information maximum likelihood strategies will be used to keep information of individuals who were randomized to one of the PAP conditions, but dropped out at any later point of the study, in the analyses. Drop-out analyses will test baseline differences between continuers and non-continuers in all variable groups using *t*-tests, chi-square tests, and logistic regressions. Identified unique differences between continuers and non-continuers (i.e., drop-out mechanisms) will be included in the imputation model (along with this study’s central variables; in case of multiple imputation) or path models (in case of full information maximum likelihood; [57]). Use of either is pre-determined by the statistical approach used to test our hypotheses.

#### Hypotheses tests

Longitudinal mediation analyses will be conducted using manifest path analyses. PrevOP-PAP-condition will be specified as a dummy-coded independent variable, accelerometer-assessed MVPA as a mediator (in minutes per week), and WOMAC scores as the primary outcome (controlling for respective baseline assessment). Dummy-coded PrevOP-MMT-conditions will be routinely tested as moderators of the association between MVPA (mediator) and WOMAC (primary outcome). If no interactions occur, PrevOP-MMT-conditions are tested as simple-effect covariates and dropped only if they are not related to the mediator or primary outcome. Further covariates are determined by results of the randomization check and bivariate correlations of assumed covariates with primary or secondary outcomes. Significance of all indirect effects will be tested using bootstrapping methods.

#### Examining intervention mechanisms

To test the assumptions of the HAPA and its extensions with social network and social exchange indicators, path analyses are employed. PrevOP-PAP condition will be specified as a dummy-coded independent variable, proposed cognitive and social intervention mechanisms as mediators (controlling for respective baseline assessments), and accelerometer-assessed PA as the outcomes (controlling for respective baseline assessment). Beyond PrevOP-MMT-conditions, further covariates are determined as outlined above. Significance of all indirect effects will be tested.

#### Additional analyses

Additional 2-level mixed model analyses will be used to examine intra-individual change (within-person) in primary and secondary outcome variables as a function of study conditions (between-person). Analyses using available self-report PA data will benefit from a larger number of - and more tightly-spaced assessments.

### Dissemination plan

The trial registration and study protocol are the first publications of this RCT. This protocol complies with SPIRIT guidelines [58]. Findings of this RCT will be published in peer-reviewed international journals and at national and international conferences. Dissemination of results in journals will comply with CONSORT guidelines [59]. Additional dissemination to the public is planned at public institutional events and via various media (website, press).

## Discussion

Using a psychological adherence program that is based on the HAPA [16] presently extended to include proposed social predictors of behavior change [30], this RCT will contribute to a better understanding of the psychological and social mechanisms of the uptake and maintenance of physical activity and their relation to disease symptoms in persons with OAK.

As reviewed throughout this study protocol, the PrevOP-PAP and this RCT have a number of strengths. However, most of these feature accompanying risks. Both are reviewed next and the discussion is closed with limitations of the RCT and an outlook.

First, the PrevOP-PAP is based on theory and makes use of a number of defined and theoretically-derived behavior change techniques [13, 16]. Amongst other benefits, this theory-centered approach facilitates the examination of active ingredients of the intervention and the cumulation of findings associated with this specified set of behavior change predictors and techniques. Theory-based approaches can thus contribute to the development of economic yet effective intervention measures and provide specific testable predictions not only for the kind of predictors playing a role in behavior change, but also for their temporal alignment in the behavior change process [16]. This provides vital information on future improvements of interventions.

A strength of this RCT is its use of objective behavioral indicator operationalizations, i.e., objective PA. Objective measures avoid many of the problems associated with common method variance or recall biases. As such, examined associations are not inflated by the fact that they are all measured as self-reports [60] and with items that are framed in a behavior-specific way and thus all directly address the behavior in question (i.e., principle of compatibility; [61]). Thus, using an objective behavioral indicator one may also reduce to some extent mere measurement effects created by a cumulation of similar sounding items that are completed and may produce behavior change themselves [62]. Accelerometers used in this study feature an additional benefit as they do not have screens and thus do not feed back the amount of PA to participants. Data have to be downloaded and processed to gain this information. This might again reduce reactive measurement and does not induce an unwanted intervention component of action control in the assessments. Drawbacks associated with the use of accelerometers, however, are also present. First, despite of the advantages described above, there have been reports of early reactive assessment effects (pertaining to baselines) when accelerometers are used [63]. Additionally, hip-worn accelerometers cannot capture activity associated with a number of specific types of PA, such as those where hips stay fairly steady (e.g., riding a bike) [64]. Relatedly, using only accelerometers, one cannot assess the type of PA a person has practiced, only time spans of different intensities of PA during the day or basic step counts can be estimated. It is for this reason that we additionally assess PA via self-report which is fraught with all the faults that have already been reviewed above.

A further strength of the RCT is its fairly long follow-up period of 24 months with a sufficient time span without interventions (12 months) to examine longer-term effects of the PrevOP-PAP on the maintenance of regular PA. Moreover, several repeated assessments allow for testing mediation processes. Again, these benefits come with certain risks, such as a relatively high expected dropout over the two years or increased participant burden due to intense intervention regimens and several repeated assessments. A number of precautions against attrition are realized within the PrevOP trial [65]. For instance, there is an active control group, participants are regularly contacted and reminded of the remaining interventions and assessments, and appointments are scheduled upfront. Many of the assessments and intervention components of both the PrevOP-PAP and PrevOP-MMT are face to face, which may also increase participant commitment.

Furthermore, computer-assisted intervention sessions facilitate standardized delivery of intervention contents, but leave room for individual interaction and tailoring intervention contents and delivery to participants’ needs.

In addition to the strengths and risks reviewed above, this RCT also has a number of limitations. An important limitation is the lack of masking or blinding within the PrevOP-PAP-I. Due to ethical and practical reasons masking is not feasible in this trial. Participants are informed about the possibility to be randomly allocated to intervention or control conditions upon inclusion and study staff are aware of the treatment they deliver. Moreover, although the PAP-I includes a network-creation component and participants are asked about social exchange processes with their sports companions and/or close others, there are no data generated by these other persons. Thus, dyadic or group effects cannot be studied in a fully reciprocal manner in this study [66]. Additionally, except for the accelerometer and assessed medical data, this study largely relies on self-report measures that are subject to recall bias and mere measurement effects as reviewed above.

As an outlook, the design and evaluation of the theory-based PrevOP-PAP are intended to become a yardstick for future development and implementation of digitalized psychological adherence programs for this population. Depending on the (to be determined) efficacy of the PrevOP-PAP and for a partially or fully digitalized implementation of the program, next steps could include the examination of different delivery modes in a factorial RCT design. Study conditions might entail graded delivery modes using different shares of personal interaction with trained study personnel to test the efficacy of a fully digitalized version of the program.

### Protocol version and trial status

This is the original study protocol as registered with the German Clinical Trials Register on 26 January 2016. No protocol modifications are planned at this time. If future protocol modifications take place, they will be reported (e.g., in the trial registry and upon publication of study results). Recruitment started in February 2016 and was continuing when the protocol was submitted for publication.

## Abbreviations

BCT: Behavior Change Techniques
CONSORT: Consolidated Standards of Reporting Trials
HAPA: Health Action Process Approach
OAK: OsteoArthritis of the Knee
PA: Physical Activity
PAP: Psychological Adherence Program
PrevOP: PREVenting the impairment of primary Osteoarthritis by high impact long-term Physical exercise regimen
PrevOP-MMT: PrevOP-Main Medical Trial
PrevOP-MMT-CTRL: PrevOP-MMT ConTRoL condition
PrevOP-MMT-HIE: PrevOP-MMT High Impact long-term physical Exercise condition
PrevOP-MMT-LIE: PrevOP-MMT Low Impact long-term physical Exercise condition
PrevOP-PAP: PrevOP-Psychological Adherence Program
PrevOP-PAP-CTRL: PrevOP-PAP ConTRoL condition
PrevOP-PAP-I: PrevOP-PAP Intervention condition
RCT: Randomized Controlled Trial
SPIRIT: Standard Protocol Items: Recommendations for Interventional Trials
T: Time
WOMAC: Western Ontario and Mc MAster universities osteoarthritis index
